# A content-based review of mobile health applications for breast cancer prevention and education: Characteristics, quality and functionality analysis

**DOI:** 10.1177/20552076241234627

**Published:** 2024-03-15

**Authors:** Stefanie Altmannshofer, Madeleine Flaucher, Milena Beierlein, Bjoern M Eskofier, Matthias W Beckmann, Peter A Fasching, Hanna Huebner

**Affiliations:** 1 Department of Gynecology and Obstetrics, 27168 Erlangen University Hospital, 9171 Friedrich-Alexander-Universität Erlangen-Nürnberg (FAU), Comprehensive Cancer Center ER-EMN, Erlangen, Germany; 2 Machine Learning and Data Analytics Lab, 88768 Friedrich-Alexander-Universität Erlangen-Nürnberg (FAU), Erlangen, Germany

**Keywords:** Breast cancer, prevention, mHealth, mobile apps, quality, MARS, rating

## Abstract

**Objective:**

Mobile Health apps could be a feasible and effective tool to raise awareness for breast cancer prevention and to support women to change their behaviour to a healthier lifestyle. The aim of this study was to analyse the characteristics and quality of apps designed for breast cancer prevention and education.

**Methods:**

We conducted a systematic search for apps covering breast cancer prevention topics in the Google Play and Apple App Store accessible from Germany using search terms either in German or in English. Only apps with a last update after June 2020 were included. The apps identified were downloaded and evaluated by two independent researchers. App quality was analysed using the Mobile Application Rating Scale (MARS). Associations of app characteristics and MARS rating were analysed.

**Results:**

We identified 19 apps available in the Google Play Store and seven apps available in the Apple App Store that met all inclusion criteria. The mean MARS score was 3.07 and 3.50, respectively. Functionality was the highest-scoring domain. Operating system, developer (healthcare), download rates and time since the last update were significantly associated with overall MARS score. In addition, the presence of the following app functions significantly influenced MARS rating: breast self-examination tutorial, reminder for self-examination, documentation feature and education about breast cancer risk factors.

**Conclusions:**

Although most of the apps offer important features for breast cancer prevention, none of the analysed apps combined all functions. The absence of healthcare professionals’ expertise in developing apps negatively affects the overall quality.

## Introduction

One in eight women will be diagnosed with breast cancer in the course of life. Breast cancer is the most common type of cancer in women with approximately 71.400 cases in 2019 in Germany.^1,2^ Although mortality is decreasing in high-income countries, mostly due to improved treatments, incidence has been steadily increasing owing to the implementation of the mammographic screening for women of 50 to 69 years.^3^ In 2018, around 2.86 million women participated in the mammographic screening programme in Germany and 17,377 of the screened women were diagnosed with breast cancer,^4^ which otherwise might have been detected later and with advanced cancer stage. This further underlines the importance of effective preventive measures.

There are multiple risk factors associated with the development of breast cancer.^5,6^ Effective and active cancer prevention and screening programmes are particularly crucial for women harbouring such risk factors. Non-genetic factors such as age, race or ethnicity, personal history of breast pathologies, high mammographic density, age at menarche and menopause are also associated with increased breast cancer risk, and education about such risk factors is crucial.^5–10^ In addition, modifiable lifestyle factors, including dietary habits, body mass index, physical activity, alcohol and tobacco consumption, use of exogenous oestrogen and reproductive history such as age at first childbirth and breastfeeding, contribute to breast cancer risk.^5–10^ The individual risk of developing breast cancer can be quantified using risk assessment tools like the Gail and Claus model.^11–13^ Various strategies exist to decrease the lifetime risk of breast cancer, including risk reduction through pharmacotherapy, surgery and dietary and nutritional interventions.^5,7,8^ To enhance the effectiveness of preventive strategies aimed at personal behaviour, the adoption of healthy habits needs to be promoted and popularized both on an individual level and a societal level.^7^

Nowadays, digital tools including mHealth apps are increasingly recognized as essential and widely used resources for provision of information and education. mHealth apps provide a range of functionalities and benefits, including tailored health information, interactive features, reminders and tracking tools and real-time monitoring of health behaviours, which are not typically available through traditional websites or other information sources.

In the context of breast cancer prevention, mHealth apps can support users in various ways: It has been shown that smartphone apps are a feasible and acceptable intervention for weight loss by tracking physical activity and nutrition,^14–16^ also specifically for breast cancer prevention in women,^17^ increasing cessation rates of tobacco and alcohol consumption,^18–20^ teaching breast self-examination (BSE),^21–23^ increasing attendance and promoting breast cancer screening programmes.^24^

However, there are little restrictions on who can develop and publish an app and regulatory organizations rarely check the validity of the health information provided in these apps.^25,26^ This has resulted in many websites^27,28^ and mHealth apps providing unclear quality of information for patients,^3,28–33^ which is particularly troublesome as patients who are not familiar with medicine topics often cannot differentiate between trustworthy, reliable and less relevant information, putting users’ health and safety at risk.^27,28,33^ While mHealth apps have a great potential to aid women in adopting healthier lifestyles, raising awareness about the significance of breast cancer prevention and offering dependable and accurate information, prior reviews about breast cancer apps are still subjected to various limitations. Some have only focused on breast cancer patients or survivors,^30,34,35^ while others conducted app store searches from other continents than Europe,^35,36^ or only analysed app content and not their quality.^30,32,34–36^ Consequently, we set out to provide a review of mobile apps available on the German Google Play or Apple App store covering particularly apps focusing on breast cancer prevention and education. This study aims to evaluate the characteristics, functionality and quality of these apps by using a multidimensional tool developed for classifying and rating the quality of mobile health apps.

## Materials and methods

This review was conducted using the Preferred Reporting Items for Systematic reviews and Meta-Analysis (PRISMA) model to remain consistent with other published systematic reviews.^37^ Furthermore, the protocol for this review was registered with the International Platform of Registered Systematic Review and Meta-analysis Protocols (INPLASY) under the registration number INPLASY202380101.^38^

### Systematic search

A systematic search of mobile apps focusing on breast cancer prevention and education accessible from Germany was conducted between March and June 2022. The search was performed using the search function within the Google Play and the Apple App store, respectively, and the search included the following search terms, either in English or in German: breast cancer, breast cancer help, breastcancer, *Brustkrebs*, *Brustkrebshilfe* and *Mammakarzinom*. We captured the app's title, developer, date of last update, price, language, categorization within the app store, download numbers and user ratings in the app store.

### App selection process

All apps matching the search terms were categorized in the following five groups if the apps are, according to the app title, description in the app store, screenshots or user comments, related to breast cancer: prevention and enlightenment, therapy, aftercare, for professionals and others. Apps addressing breast cancer social network apps or diary apps were categorized in “Others”. Apps were excluded and defined as false hit based on the following criteria: (a) no relation to breast cancer, e.g. apps made for other types of cancer; (b) had a relation to breast cancer but did not fit in one of the above groups, e.g. apps without a benefit for the user itself like wallpaper apps; and (3) were not available in either English or German.

Duplicates within the results for the different search terms were removed, and only apps that were part of the prevention and enlightenment category, and were last updated after June 2020, were downloaded for further evaluation. Only duplicates within each of the app stores (Google or Apple) were removed. Apps available within both app stores were included for each app store individually. Downloaded apps were examined for their technical aspects including share on social media, an app community, registration necessary, in-app purchases, show advertisements and data management, prevention-related implemented functions like BSE tutorials, reminders for self-examination, documentation feature to note findings, information about breast cancer risk factors, mammography and screening programs, citation of scientific literature and reports of patients who have already undergone breast cancer therapy and rated using the Mobile Application Rating Scale (MARS).^39^ Technical subgroup definition was inspired by Bardus et al.^40^ Technical and prevention-related implemented features were captured and displayed as tables.

#### Functionality behind paywalls was not analysed

Two independent reviewers tested each app in a real-world setting. The apps were rated and reviewed in iOS 16.1 with an iPhone 12 and on Android 9 with a Samsung Galaxy S8. Second rater used an iPhone SE (iOS 15.6.1) and a Google Pixel 6 (Android 13).

### Rating using the Mobile Application Rating Scale

Two independent reviewers rated each app using the MARS. The MARS is a simple, reliable and objective tool for analysing, classifying and evaluating the quality of mobile applications, which is also used in similar publications.^39–41^ It includes an app classification section to collect descriptive and technical information, an app quality rating section including 23 items in five sections:

1. Engagement – is the app fun, interesting, customizable, interactive (e.g. sends alerts, messages, reminders and feedback and enables sharing) and well-targeted to the audience?
2. Functionality – app functioning, easy to learn, navigation, flow logic and gestural design of the app.
3. Aesthetics – graphic design, overall visual appeal, colour scheme and stylistic consistency.
4. Information – is the app containing high-quality information (e.g. text, feedback, measures and references) from a credible source?
5. Satisfaction – app subjective quality

And an app-specific section to assess the perceived impact of the app on the user. Each item uses a 5-point scale (1, inadequate; 2, poor; 3, acceptable; 4, good; 5, excellent; from 1, strongly disagree to 5, strongly agree, respectively). Item 19 of MARS was excluded, since none of the reviewed apps was trialled before. The mean value between the values of both reviewers of each item in a section was calculated, and based on these mean values of each item, the mean values and standard deviations of the quality subscales (engagement, functionality, aesthetics, information and satisfaction) were calculated.^42^ The mean overall score represents the mean overall objective subscales (engagement, functionality, aesthetics and information), while the mean value of satisfaction refers to the subjective quality.

The scores were presented as means ± standard deviation (SD). Interrater reliability was calculated using Cohen's kappa.

### Statistical analyses

Association of MARS scores with app characteristics and breast cancer prevention-specific app features, respectively, were evaluated using the Mann–Whitney-U test to an exact significance level of p < 0.05 using the IBM SPSS Statistics (Version 28.0.0.0). The interrater reliability coefficient Cohen κ was calculated using *R* (Version 4.2.2).^43^

Given our review's focus on app evaluation within app stores, traditional risk of bias assessment methods were not directly applicable; instead, we conducted a systematic analysis of app attributes, functionalities and user feedback to thoroughly assess their features and characteristics.

## Results

### Systematic search

**Figure 1.**
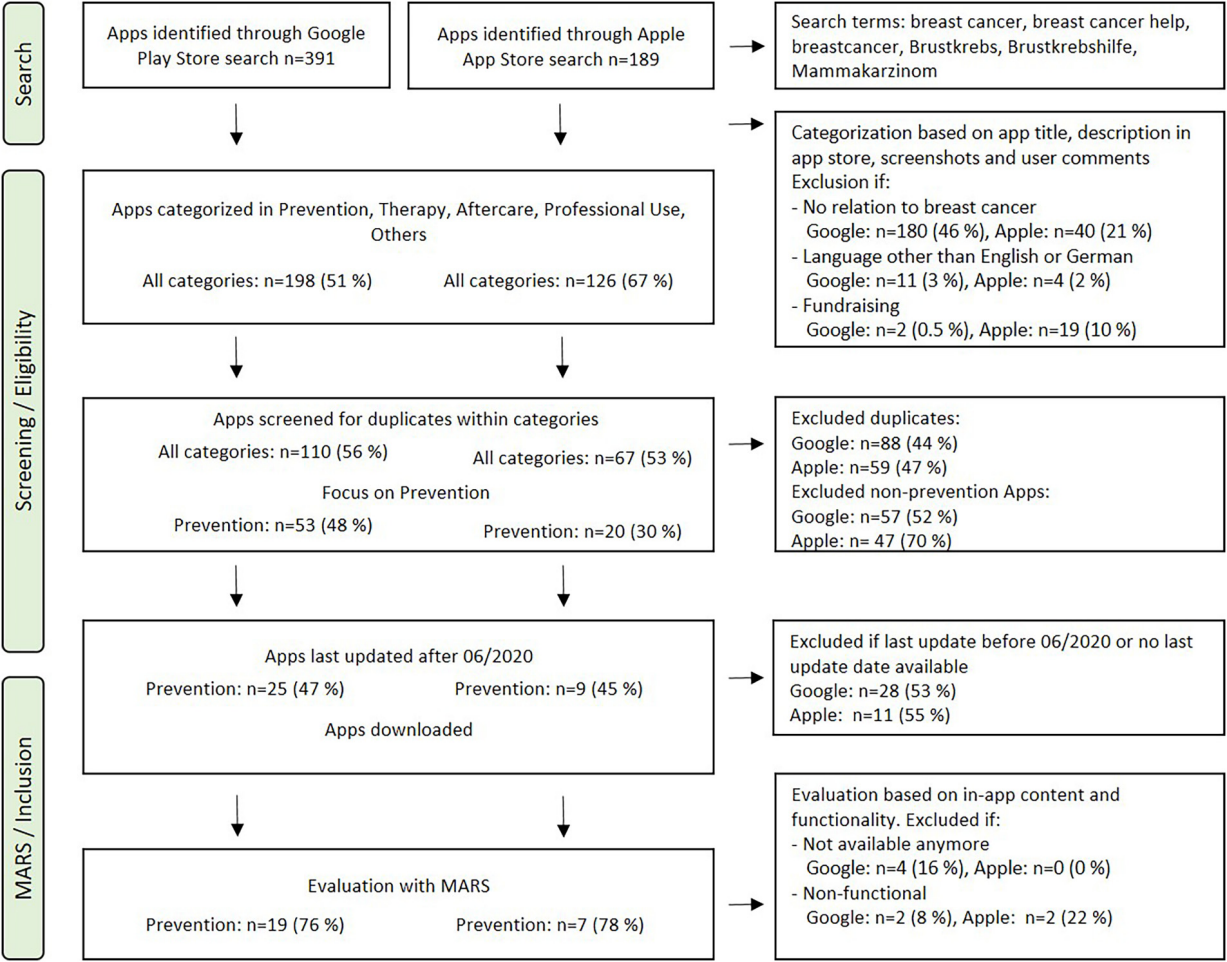
Systematic search for breast cancer prevention apps on Google Play and the Apple App Store.

The Google Play and Apple App Store search identified 391 and 189 apps, respectively (Figure 1). Apps were categorized as described above, 198 and 126 apps matched the described groups, and 193 and 63 were false hits, since they had no relation to breast cancer, did not fit in one of the categories or were not available in either English or German (Appendix 1). An app was assigned to the category “prevention”, if either the app title, app description in the app store, screenshots or user comments indicate that the app has preventive features such as a self-examination tutorial, reminders for self-examination, information about breast cancer risk factors and information about mammography and screening programmes. After excluding duplicates, 110 apps remained in the Google Play Store category, 53 of which were further categorized as apps associated with the topic “prevention”. Within the Apple App Store category, 67 apps remained and 20 could be assigned to the topic “prevention”. Apps not assigned to the category “prevention” were excluded. Apps last updated before June 2020 were excluded (n = 38). The criterion of including only apps updated within the last 2 years was employed to ensure the present-day relevance and accuracy of the analysed apps. Regular updates in the rapidly evolving landscape of health-related apps are indicative of ongoing development, medical accuracy and up-to-date IT and data security standards, aligning with best practices for evaluating the usability and functionality of such apps. 25 apps on Android and nine on iOS were included for further review and evaluation with MARS (Figure 1).

At the time of download (October 2022), four apps (“Breast cancer – Symptoms & Prevention Tips”, “Breast Cancer, Causes and Prevention”, “Breast Cancer” developed by Active App Builder and “Breast Care – Take Care of Breasts the Right Way”) were no longer available. One app (“Bscan – Breast Cancer Screening”) was not available in English or German, although information on Google Play suggested an English app. These apps were excluded from further evaluation. Two apps (“Brexa” and “Know your lemons breast health app”) only worked for one rater, since the apps crashed when trying to use them on iOS16.1 with an iPhone 12 and on Android 9 with a Samsung Galaxy S8 (Figure 1).

Overall, we reviewed 21 different apps. 19 of those apps were available in the Google Play Store and seven in the Apple App Store. Five apps were available on both platforms and thus were evaluated using both operating systems, Android and iOS.

### Features of breast cancer prevention apps

General characteristics of the evaluated apps are shown in Table 1. All apps were free for download. Most of the apps were assigned to the app store category “Health&Fitness” (43% of the apps from the Google Play Store and 58% from the Apple App Store) and Medicine (26% of the apps from the Google Play Store and 14% from the Apple App Store). 68% of the Android exclusive apps and all of the Apple exclusive apps were lastly updated within 1 year. There were only three apps in German on Google Play Store, of which one is also available in English; two German Apps were identified in the Apple App Store. Download rates were only accessible on Google Play, which were in a range from 1+ to 50,000+ downloads. The majority of the apps found on Google Play were developed by non-health organizations (68%), while in the Apple App store, 71% were developed by a health organization.

**Table 1.** General characteristics of the apps.

Characteristics	Google, n (%)	Apple, n (%)
**N**	19 (73)	7 (27)
**Cost**		
No	19 (73)	7 (27)
Yes	0 (0)	0 (0)
**Category**		
Medicine	5 (26)	1 (14)
Health&Fitness	9 (47)	4 (58)
Education	2 (11)	1 (14)
Lifestyle	1 (0)	1 (14)
Entertainment	2 (11)	0 (0)
**Date of the last update**		
<1 year	13 (68)	7 (100)
>1 year	6 (32)	0 (0)
**Language**		
English	16 (84)	5 (71)
German	2 (11)	2 (29)
English and German	1 (5)	0 (0)
**Downloads** ^a^		
<100	3 (16)	-
<1.000	5 (26)	-
<10.000	5 (26)	-
<100.000	6 (32)	-
≥100.000	-	-
**Developer**		
Health organization	5 (26)	5 (71)
Others	13 (68)	2 (29)

^a^ Number of downloads was only available for Google Play Store.

Technical features of the reviewed apps are summarized in Appendix 2 and preventive functionalities are depicted in Table 2.

Technical subgroups were inspired by Bardus et al.^40^:

- Social media: Allows sharing on social media
- App community: Has an app community for sharing information with other app users
- In-app purchase: Payments required for full app version or add-ins
- Registration: Registration necessary to have access to the app
- Advertisements: Shows advertisements for products or websites either via pop-ups or banners
- Data management: Needs agreement on a data consent form or informs the user about the apps data management like third-party involvement

**Table 2.** List of identified breast cancer preventive functionalities with number of occurrences for each distribution platform.

App store	Breast self-examination	Notification	Documentation	Mammography	Risk factors	Literature	Reports of patients	Sum of features
Google Play Store (n (%))	8 (42)	3 (16)	2 (11)	6 (32)	1 (5)	4 (21)	0 (0)	24 (18)
Apple App Store (n (%))	1 (50)	1 (50)	0 (0)	1 (50)	0 (0)	0 (0)	1 (50)	4 (29)
Both Platforms (n(%))	5 (100)	4 (80)	3 (60)	3 (60)	2 (40)	3 (60)	3 (60)	23 (66)
Total^*^ (n (%))	14 (67)	8 (38)	5 (24)	10 (48)	13 (62)	7 (33)	4 (19)	61 (41)

* Number of differing apps: n = 21. Apps available on both platforms were counted as one app.

Preventive feature subgroups were defined according to found functionalities in all apps:

- Breast self-examination: Has a tutorial for BSE either in written or visual form included
- Notification: Reminds the user of the next BSE or appointment for mammography
- Documentation: Allows documentation of BSE results or menstrual cycle tracking
- Mammography: Gives information about mammography in general and about mammography and screening programmes
- Risk factors: Gives information about breast cancer risk factors
- Literature: Shares the scientific references for the information about breast cancer or shares links for further reading
- Reports of patients: Shares stories of patients who already had breast cancer or are still undergoing therapy

Tables with each app and its implemented technical and preventive features listed are shown in Appendix 2 and Appendix 3, respectively.

None of the analysed apps had an app community; four (19%) apps allowed sharing on social media (Appendix 2). Only one required an in-app purchase for the full version. Registrations were necessary for two (10%) apps, and for two other apps, it was possible either to use them as a guest or to create an account. However, those apps were not customizable when logged in as a guest. One app requested permission for app tracking.

The majority of apps (67%, n = 14) had a tutorial for a BSE implemented, either in written form or via animations or videos (Table 2). Eight (38%) of these had also a notification function to remind the user for the self-examination in a weekly, monthly, period-dependent or independent recurrence. This function did not work for one app (“Stan Swasthya”). A diary function to note and track their findings and their menstrual cycle was implemented in 24% (n = 5) of the apps that had a tutorial for a BSE. 48% of the apps (n = 10) had information about mammography and screening programmes and 62% (n = 13) inform about risk factors for breast cancer, but only 33% (n = 7) of the apps shared their references or linked scientific literature for further information. Reports of patients who have already had breast cancer or are still undergoing therapy were included in 19% (n = 4) of the apps.

### App quality

Table 3 shows the subscale and overall scores of apps rated with MARS. The mean overall MARS score was 3.07 (SD = 0.51) out of 5 ranging from 2.28 (SD = 1.03, “Brustkrebs”) to 4.18 (SD = 0.54, “Keep a Breast”) for apps available in the Google Play Store. The mean overall MARS score for apps available in the Apple App Store was 3.50 (SD = 0.36) ranging from 3.12 (SD = 1.04, “empower breast AR”) to 4.18 (SD = 0.54, “Keep a Breast”). Only two apps (“Keep a Breast” and “Dear Mamma”) met or bet the minimum acceptability score of 3.0 Satisfaction, which is the only complete subjective subscale that is not included in the overall MARS score. Functionality was the highest-scoring domain (Mean = 3.91 (SD = 0.45) for Google and Mean = 3.98 (SD = 0.17) for Apple) followed by Information (Mean = 2.97 (SD = 0.71) for Google and Mean = 3.69 (SD = 0.36) for Apple; Table 3).

**Table 3.** MARS scores of the evaluated apps.

		Objective scale					Subjective scale
	App	Engagement	Functionality	Aesthetics	Information	Overall	Satisfaction^*^
Google Play Store	Keep a Breast	3.90 (0.20)	4.25 (0.43)	4.83 (0.24)	4.00 (0.00)	4.18 (0.54)	3.38 (0.41)
	Dear Mamma	3.90 (0.66)	3.75 (0.43)	3.17 (0.24)	4.08 (0.61)	3.81 (0.63)	3.00 (1.22)
	breastcare - Brustbewusstsein	3.00 (0.63)	4.13 (0.22)	3.67 (0.47)	4.20 (0.40)	3.74 (0.69)	2.88 (1.14)
	Selbstuntersuchung der Brust	3.20 (0.40)	4.25 (0.43)	4.67 (0.00)	3.25 (1.15)	3.69 (0.96)	2.75 (1.03)
	BREAST TEST	3.10 (1.11)	4.75 (0.43)	3.50 (0.41)	3.40 (0.80)	3.65 (1.01)	2.13 (0.74)
	Boot out breast cancer	3.10 (0.49)	4.00 (0.00)	3.33 (0.47)	3.50 (0.45)	3.47 (0.53)	2.13 (0.74)
	Breast Cancer (Focus)	2.10 (1.11)	4.00 (0.00)	3.17 (0.62)	3.40 (1.24)	3.12 (1.18)	1.63 (0.65)
	Beat Cancer	3.30 (0.98)	3.88 (0.54)	2.67 (0.94)	2.60 (1.16)	3.12 (1.08)	2.00 (0.71)
	Breast Cancer Survival Guide for Patients	2.50 (0.89)	3.75 (0.43)	2.33 (0.47)	3.30 (1.17)	3.00 (1.01)	2.00 (0.71)
	In the PINK PH	2.30 (1.08)	4.00 (0.00)	3.00 (0.00)	2.90 (0.49)	3.00 (0.89)	1.25 (0.43)
	Stan Swasthya	2.60 (0.80)	3.50 (0.61)	2.83 (0.85)	3.00 (1.41)	2.97 (1.05)	1.25 (0.25)
	Young Breast Cancer Warriors	2.00 (1.10)	3.75 (0.43)	2.67 (0.94)	3.40 (1.20)	2.94 (1.21)	1.38 (0.41)
	NextGen Breast Examination: Breast Cancer	2.50 (0.63)	2.75 (0.43)	3.00 (0.82)	2.40 (0.49)	2.62 (0.63)	1.88 (0.54)
	Breast Cancer (Guide)	1.50 (1.00)	4.75 (0.43)	1.50 (0.41)	2.50 (0.94)	2.56 (1.54)	1.50 (0.50)
	Breast cancer (Yazan Halawi)	2.00 (1.10)	4.25 (0.43)	1.83 (0.24)	2.13 (0.89)	2.56 (1.26)	1.50 (0.50)
	Brustkrebs - Bewusstsein	1.90 (0.92)	3.75 (0.43)	1.83 (0.24)	2.50 (1.00)	2.50 (1.07)	1.00 (0.00)
	Breast Health Care (BHM)	2.40 (0.49)	3.25 (0.25)	2.17 (0.62)	1.80 (0.75)	2.38 (0.78)	1.00 (0.00)
	Breast Disorder	1.50 (0.63)	3.75 (0.43)	2.17 (0.24)	2.10 (1.02)	2.32 (1.08)	1.50 (0.50)
	Brustkrebs	1.80 (0.75)	3.75 (0.43)	1.67 (0.24)	2.00 (0.82)	2.28 (1.03)	1.00 (0.00)
	All [Mean (SD)]	2.56 (0.71)	3.91 (0.45)	2.84 (0.90)	2.97 (0.71)	3.07 (0.51)	1.85 (0.70)
Apple App Store	Keep a Breast	3.90 (0.20)	4.25 (0.43)	4.83 (0.24)	4.00 (0.00)	4.18 (0.54)	3.38 (0.41)
	Dear Mamma	3.90 (0.66)	3.75 (0.43)	3.17 (0.24)	4.08 (0.61)	3.81 (0.63)	3.00 (1.22)
	breastcare - Brustbewusstsein	3.00 (0.63)	4.13 (0.22)	3.67 (0.47)	4.20 (0.40)	3.74 (0.69)	2.88 (1.14)
	Boot out breast cancer	3.10 (0.49)	4.00 (0.00)	3.33 (0.47)	3.50 (0.45)	3.47 (0.53)	2.13 (0.74)
	BreastAware - BCI	2.80 (0.75)	4.00 (0.00)	3.00 (0.00)	3.25 (0.92)	3.25 (0.79)	1.63 (0.48)
	empower Breast AR	2.60 (0.80)	4.00 (0.00)	2.33 (0.47)	3.40 (1.24)	3.12 (1.04)	2.25 (0.83)
	All [Mean (SD)]	3.04 (0.63)	3.98 (0.17)	3.29 (0.75)	3.69 (0.36)	3.50 (0.36)	2.38 (0.68)

Values expressed as mean (standard deviation) of the items of each quality score (sorted by highest overall score for Google Play Store and Apple App Store).

* Satisfaction is not included in the overall score calculation.

Comparison by app distribution platforms (Apple App Store and Google Play Store) revealed a mean Information MARS score of 2.97 (SD = 0.73) for apps from Google Play Store (n = 17) and 3.69 (SD = 0.39) for apps from the Apple App Store (n = 7) resulting in a statistically significant difference (p = 0.022; Table 4). Apps which had been developed by health organizations (N = 11) obtained better scores in all categories compared to those from other developers (n = 15, p < 0.001). Apps that had reported numbers of downloads above 10,000 had significantly higher MARS scores for the categories Engagement (p = 0.009), Aesthetics (p = 0.029), Satisfaction (p = 0.005) and the overall MARS score (p = 0.022) compared to apps with lower download numbers (Table 4). Similarly, apps that were recently updated (<1 year) had a significantly higher MARS score for Information category (p = 0.009, Table 4).

**Table 4.** Association of mobile app rating scale evaluation with app characteristics.

			Objective scale					Subjective scale
		n	Engagement	Functionality	Aesthetics	Information	Overall	Satisfaction
Operating system	Android	19	2.56 (0.73)	3.91 (0.47)	2.84 (0.92)	2.97 (0.73)	3.05 (0.57)	1.85 (0.72)
	iOS	7	3.04 (0.69)	3.98 (0.18)	3.29 (0.81)	3.69 (0.39)	3.50 (0.44)	2.38 (0.74)
	p-value		0.169	0.611	0.231	0.022	0.063	0.083
Developer	HO	11	3.27 (0.55)	4.09 (0.27)	3.59 (0.65)	3.74 (0.44)	3.66 (0.36)	2.53 (0.71)
	Others	15	2.26 (0.54)	3.81 (0.46)	2.50 (0.78)	2.75 (0.59)	2.81 (0.39)	1.60 (0.50)
	p-value		<0.001	0.047	<0.001	<0.001	<0.001	0.001
Number of downloads	<10,000	13	2.27 (0.59)	3.90 (0.37)	2.50 (0.69)	2.77 (0.71)	2.84 (0.45)	1.54 (0.54)
	≥10,000	6	3.18 (0.63)	3.92 (0.68)	3.58 (0.98)	3.41 (0.61)	3.49 (0.57)	2.52 (0.61)
	p-value		0.009	0.701	0.029	0.152	0.022	0.005
Last update	<1year ago	20	2.78 (0.74)	3.95 (0.41)	3.10 (0.90)	3.37 (0.72)	3.29 (0.57)	2.09 (0.75)
	≥1 year ago	6	2.38 (0.77)	3.86 (0.80)	2.50 (0.71)	2.47 (0.54)	2.78 (0.51)	1.67 (0.42)
	p-value		0.355	0.656	0.157	0.009	0.062	0.324

Values expressed as mean (standard deviation) of the items of each quality score or number. HO: Health organization.

In addition, MARS scores were compared between apps with or without certain features relevant for prevention of breast cancer (Table 5). Significant differences were observed for the MARS scores Engagement, Aesthetics, Information and Satisfaction as well as the overall MARS score, between apps that include a BSE tutorial (Overall, p = 0.002), a reminder (Overall, p < 0.001) or documentation (Overall, p = 0.003) function or provide education about breast cancer risk factors (Overall, p = 0.001) and those that lack these features (Table 5).

**Table 5.** Association of mobile app rating scale evaluation with breast cancer prevention specific app features.

			Objective scale					Subjective scale
		n	Engagement	Functionality	Aesthetics	Information	Overall	Satisfaction
BSE	Yes	19	2.93 (0.70)	3.97 (0.44)	3.25 (0.84)	3.42 (0.57)	3.36 (0.36)	2.21 (0.72)
	No	7	2.04 (0.37)	3.82 (0.31)	2.17 (0.50)	2.48 (0.66)	2.61 (0.36)	1.41 (0.46)
	p-value		0.004	0.364	0.002	0.006	0.002	0.018
N	Yes	12	3.29 (0.48)	4.06 (0.32)	3.67 (0.71)	3.71 (0.43)	3.66 (0.35)	2.55 (0.68)
	No	14	2.17 (0.48)	3.81 (0.45)	2.36 (0.53)	2.70 (0.59)	2.75 (0.32)	1.52 (0.40)
	p-value		<0.001	0.076	<0.001	<0.001	<0.001	<0.001
D	Yes	8	3.43 (0.54)	3.94 (0.58)	3.65 (0.75)	3.62 (0.57)	3.65 (0.50)	2.63 (0.62)
	No	18	2.36 (0.55)	3.92 (0.33)	2.66 (0.80)	2.96 (0.70)	2.96 (0.46)	1.71 (0.62)
	p-value		<0.001	0.605	0.003	0.019	0.003	0.002
M	Yes	13	2.49 (0.60)	3.84 (0.23)	2.71 (0.66)	3.06 (0.79)	3.01 (0.52)	1.76 (0.65)
	No	13	2.88 (0.83)	4.02 (0.53)	3.22 (1.05)	3.27 (0.66)	3.33 (0.59)	2.22 (0.79)
	p-value		0.204	0.113	0.243	0.687	0.139	0.125
RF	Yes	15	2.26 (0.54)	3.89 (0.35)	2.52 (0.68)	2.90 (0.75)	2.88 (0.46)	1.61 (0.60)
	No	11	3.27 (0.54)	3.98 (0.49)	3.56 (0.84)	3.53 (0.50)	3.57 (0.46)	2.51 (0.61)
	p-value		<0.001	0.259	0.002	0.036	0.001	<0.001
L	Yes	10	2.90 (0.70)	4.06 (0.31)	3.38 (0.89)	3.54 (0.55)	3.45 (0.51)	2.27 (0.82)
	No	16	2.56 (0.75)	3.84 (0.45)	2.70 (0.83)	2.93 (0.73)	3.00 (0.55)	1.82 (0.67)
	p-value		0.286	0.201	0.077	0.053	0.097	0.241

Values expressed as mean (standard deviation) or numbers. BSE: Breast self-examination tutorial either in written or/and in visual (pictures, animations, videos) form. N: Push-notification to remind for self-examination and healthcare appointments. D: Documenting results of self-examination, physical condition and menstrual cycle. M: General information about mammography. RF: General information about breast cancer risk factors. L: References for evidence-based information and literature.

The mean κ coefficient for the five MARS domains was 0.79 (Table 6). A coefficient between 0.61 and 0.80 indicates a substantial agreement between the two raters. The only two items with a score below 0.61 were accuracy of the app description in the app store and subjective overall star rating of the app (Table 6).

**Table 6.** Kappa score and interrater reliability for the mobile app rating scale domains.

Domain	Cohen κ	Agreement
**Engagement**		
Entertainment	κ = 0.93	Almost perfect or perfect
Interest	κ = 0.85	Almost perfect or perfect
Customization	κ = 0.72	Substantial
Interactivity	κ = 0.93	Almost perfect or perfect
Target Group	κ = 0.65	Substantial
**Functionality**		
Performance	κ = 0.86	Almost perfect or perfect
Ease of use	κ = 0.75	Substantial
Navigation	κ = 0.64	Substantial
Gestural design	κ = 0.91	Almost perfect or perfect
**Aesthetics**		
Layout	κ = 0.80	Substantial
Graphics	κ = 0.64	Substantial
Visual appeal	κ = 0.70	Substantial
**Information**		
Accuracy of the app in the description in app store	κ = 0.52	Moderate
Goals	κ = 1.00	Almost perfect or perfect
Quality of information	κ = 0.91	Almost perfect or perfect
Quantity of information	κ = 0.61	Substantial
Visual information	κ = 0.70	Substantial
Credibility	κ = 1.00	Almost perfect or perfect
**Subjective quality**		
Would you recommend this app to people who might benefit from it?	κ = 0.86	Almost perfect or perfect
Would you pay for this app?	κ = 0.86	Almost perfect or perfect
How many times would you think you would use this app in the next 12 months if it was relevant for you?	κ = 1.00	Almost perfect or perfect
What is your overall star rating of the app?	κ = 0.46	Moderate

## Discussion

This study represents the first analysis and review of mobile apps focused on breast cancer prevention and education available through either the German Google or the German Apple app platforms. We searched the app stores, categorized the apps and used the MARS tool to rate those focused on breast cancer prevention and education.

Our findings show that “Keep a Breast” is the best breast cancer prevention app in the Google and Apple App Stores, based on the highest MARS scores. “Dear Mamma” and “breastcare – Brustbewusstsein” scored highest in the information section, thus being an enrichment for breast cancer prevention strategies. Regarding all analysed MARS domains, functionality was the highest scoring MARS domain, indicating that developers pay attention to create an easy-to-use, internally consistent and logical app. This aligns with the conclusions drawn from earlier review studies, which emphasized the significance of usability and functionality in app development.^40,41,44^ The overall objective score of all identified apps only narrowly reached the minimum acceptability score and highlights the need for improvement. Others have similarly identified many apps with just about acceptable scores among breast cancer prevention apps.^41,45^

Most of the reviewed apps provide tutorials for BSE, reminders and general information, but none combined all these functions. More than 60% of the apps originated from sources with questionable legitimacy or trustworthiness, none were trialled before release and, although the information section achieved the second-highest MARS score in our study, the mean achieved score has to be considered as low. Almost half of the apps (43%) were below the minimum acceptability score within the information section. In addition, only 33% shared their references, indicating a lack of academic and medical input. A general deficiency regarding trustworthy references within eHealth apps was also demonstrated by others.^30,32,36,44^ This goes in line with the absence of the involvement of healthcare professionals throughout the app development and raises concerns regarding the credibility of medical information within such apps. Involving medical personnel in mHealth app development is essential in order to provide a comprehensive expertise on the relevant topics, yet this rarely occurs.^30,36^ Our data indicates that apps developed in collaboration with healthcare professionals are of higher quality compared to others, highlighting the need for medical expertise and knowledge. Particularly in areas such as breast cancer prevention and early detection, an active participation from healthcare organizations and governmental agencies is required to ensure reliable, up-to-date evidence-based and scientific education within eHealth apps.^28,31,33^

It has been shown that mHealth apps can be effective and well-received tools for promoting weight loss by tracking physical activity, diet and nutrition, to increase cessation rates for alcohol or tobacco consumption, to teach practices like BSE or to remind for regular screening appointments.^17–24^ However, none of the reviewed apps included other preventive strategies besides BSE practice and provision of information about risk factors and mammography screening. Additionally, none of the apps allowed for tracking of physical activity, diet or nutrition. As the implementation of tracking features is associated with a big workload and, thus, high costs, the financing of such projects is problematic. Since the German regulations does not allow prevention apps to be labelled as a regular health insurance service, developers of prevention mHealth apps are not able to receive health insurance funding.^46^ However, a study investigating the cost-effectiveness of lifestyle-related interventions for primary prevention of breast cancer found evidence that diet and physical activity-related interventions were cost-effective.^47^ Moreover, e-health interventions for treatment of adolescent overweight and obesity demonstrated very good cost-effectiveness potential.^48^ This underscores the significant potential of preventive health apps and highlights the necessity of a comprehensive discussion regarding funding mechanisms, involving both policy makers and insurance entities.

Also, none of the apps could be personalized based on individual risk factors such as age, family history or lifestyle habits. Therefore, all analysed apps lacked personalized prediction of breast cancer risk and personalized suggestions for cancer prevention. Personalization is essential for mHealth apps designed to support patients affected by more complex diseases, and customization should be fully configurable to meet individual needs.^49,50^ To keep users engaged, it is suggested to enhance interactivity between the user and the app.^49–52^

Despite the rigorous methodology employed in this study, some limitations should be noted. First, the search was limited to the Google Play Store and Apple App Store, and apps from other sources may have been missed. Furthermore, since the search was only in Germany, the findings might not apply elsewhere. Due to the study including apps in both German and English languages, but the app search being conducted within a German context only, there is a possibility that not all English-language apps were comprehensively captured, which might introduce an additional potential source of bias. Second, we only considered apps updated after June 2020, possibly excluding relevant older apps. However, it can be assumed that older apps might not be up-to-date regarding provision of information, are mostly not suitable for current versions of smartphone operating systems and would show even worse MARS rating. Third, the study only assessed app quality using the MARS tool, which may not capture all aspects of app functionality and user experience. And lastly, our review focused on publicly available apps only, in order to provide a comprehensive overview of apps which might be used by the German population. Therefore, we did not include apps meant for research or limited to specific healthcare providers.

## Conclusions

In conclusion, this study showed that the majority of apps designed for breast cancer prevention offer tutorials for BSE, reminders or general information on breast cancer risk factors and screening. However, none of the apps comprised tracking functions or breast cancer risk assessment tools. Quality assessment using the MARS tool revealed poor quality of total content and information provided by these apps, indicating a lack of comprehensive, trustworthy and evidence-based information, and little involvement of medical personnel in their development. The reviewed apps also lacked personalized risk prediction and suggestions for cancer prevention. Personalization, interactivity and involvement of healthcare professionals are recommended to improve the quality and credibility of such mHealth apps and should be considered for future developments.

## Appendix

**Appendix 1. Categorization of apps found by systematic search in the Google Play Store and Apple App Store. Values expressed as numbers.**

App store	Search string	Found apps	False hits	Prevention /enlightenment	Therapy	Aftercare	Professional use	Others
Google Play Store	Breastcancer	243	141	51	16	2	5	29
	Breast cancer	29	1	13	5	1	1	8
	Breast cancer help	28	1	13	6	1	1	6
	Brustkrebs	30	15	6	5	1	1	2
	Brustkrebs Hilfe	29	17	2	8	1	0	2
	Mammakarzinom	30	18	4	4	0	1	3
Apple App Store	Breastcancer	63	18	15	8	14	3	5
	Breast cancer	74	24	17	9	6	2	16
	Breast cancer help	4	0	1	1	0	0	2
	Brustkrebs	39	20	4	9	2	0	4
	Brustkrebs Hilfe	2	0	1	1	0	0	0
	Mammakarzinom	7	1	0	4	0	0	2

## Appendix

**Appendix 2. List of identified technical functionalities with number of occurrences.**

	App	Social media	In-app purchase	Registration	Advertisements	Data management
Google Play Store	Beat Cancer - 100 Cancer Prevention Tips					
	Breast cancer (Focus)		x			
	Breast Cancer (Guide)				x	
	Breast Cancer (Yazan Halawi)					
	Breast Cancer Survival Guide for Patients				x	
	Breast Disorder					
	Breast Health Care (BHM)			x		
	BREAST TEST					
	Brustkrebs				x	
	Brustkrebs - Bewusstsein (Breast Cancer)				x	
	In The Pink PH					
	NextGen Breast Examination: Breast Cancer			x		
	Selbstuntersuchung der Brust					
	Stan Swasthya					
Apple App Store	breast Aware - BCI					
	empower Breast AR					
Both Platforms	Boot Out Breast Cancer					
	breastcare - Brustbewusstsein	x				
	DearMamma	x				x
	Keep a breast	x		x	x	
	Young Breast Cancer Warriors	x		x		
Google Play Store (n (%))		4 (21)	1 (5)	2 (10)	4 (21)	0 (0)
Apple App Store (n (%))		0 (0)	0 (0)	0 (0)	0 (0)	0 (0)
Both Platforms (n (%))		4 (80)	0 (0)	2 (40)	1 (20)	1 (20)
Total^*^ (n (%))		4 (19)	1 (5)	4 (19)	5 (24)	1 (5)

* Number of differing apps: N = 21. Apps available on both platforms were counted as one app.

## Appendix

**Appendix 3.** List of identified breast cancer preventive functionalities sorted by number of occurrences for each distribution platform.

	App	Breast self-examination	Notification	Documentation	Mammography	Risk Factors	Literature	Reports of patients	Sum of features
	Beat Cancer - 100 Cancer Prevention Tips	x			x	x	x		4/7
	BREAST TEST	x	x	x			x		4/7
	Breast Cancer Survival Guide for Patients	x			x	x	x		4/7
	In The Pink PH	x				x	x		3/7
	Stan Swasthya	x	x*			x			3/7
	Breast Health Care (BHM)				x	x			2/7
	Breast Cancer (Guide)	x				x			2/7
	Breast Disorder				x	x			2/7
	NextGen Breast Examination: Breast Cancer	x		x					2/7
	Brustkrebs - Bewusstsein (Breast Cancer)				x	x			2/7
	Brustkrebs				x	x			2/7
	Selbstuntersuchung der Brust	x	x						2/7
	Breast cancer (Focus)					x			1/7
	Breast Cancer (Yazan Halawi)					x			1/7
Apple	breast Aware - BCI	x	x		x			x	4/7
	empower Breast AR								0/7
Both Platforms	breastcare - Brustbewusstsein	x	x		x	x	x	x	6/7
	Keep a breast	x	x	x			x	x	5/7
	Young Breast Cancer Warriors	x			x	x	x	x	5/7
	Boot Out Breast Cancer	x	x	x	x				4/7
	DearMamma	x	x	x					3/7
Google Play Store (n (%))		8	3	2	6	1	4	0	24
		(42)	(16)	(11)	(32)	(5)	(21)	(0)	(18)
Apple App Store (n (%))		1	1	0	1	0	0	1	4
		(50)	(50)	(0)	(50)	(0)	(0)	(50)	(29)
Both Platforms (n (%))		5 (100)	4 (80)	3 (60)	3 (60)	2 (40)	3 (60)	3 (60)	23 (66)
Total^**^ (n (%))		14	8	5	10	13	7	4	61
		(67)	(38)	(24)	(48)	(62)	(33)	(19)	(41)

* Notification feature was implemented, but did not work.

** Number of differing apps: N = 21. Apps available on both platforms were counted as one app.

## References

[1] Robert Koch Institut. Krebs gesamt, https://www.krebsdaten.de/Krebs/DE/Content/Krebsarten/Krebs_gesamt/krebs_gesamt_node.html (accessed 22.11. 2022).

[2] Robert Koch Institut. Brustkrebs, https://www.krebsdaten.de/Krebs/DE/Content/Krebsarten/Brustkrebs/brustkrebs_node.html (accessed 22.11. 2022).

[3] Narod SA Iqbal J Miller AB . Why have breast cancer mortality rates declined? J Cancer Policy 2015; 5: 8–17.

[4] Kooperationsgemeinschaft Mammographie. Jahresbericht Evaluation 2018. Berlin: Deutsches Mammographie-Screening-Programm, 2020.

[5] Britt KL Cuzick J Phillips KA . Key steps for effective breast cancer prevention. Nat Rev Cancer 2020; 20: 417–436. 32528185 10.1038/s41568-020-0266-x

[6] Mahoney MC Bevers T Linos E , et al. Opportunities and strategies for breast cancer prevention through risk reduction. CA Cancer J Clin 2008; 58: 347–371. 18981297 10.3322/CA.2008.0016

[7] Sauter ER . Breast cancer prevention: current approaches and future directions. Eur J Breast Health 2018; 14: 64–71. 29774312 10.5152/ejbh.2018.3978 PMC5939980

[8] Harvie M Howell A Evans DG . Can diet and lifestyle prevent breast cancer: what is the evidence? Am Soc Clin Oncol Educ Book 2015; 35: e66–e73. 10.14694/EdBook_AM.2015.35.e66 25993238

[9] Stickeler E Aktas B Behrens A , et al. Update breast cancer 2021 part 1 – prevention and early stages. Geburtshilfe Frauenheilkd 2021; 81: 526–538. 34035547 10.1055/a-1464-0953 PMC8137274

[10] Fehm TN Welslau M Muller V , et al. Update breast cancer 2022 part 3 – early-stage breast cancer. Geburtshilfe Frauenheilkd 2022; 82: 912–921. 36110894 10.1055/a-1912-7105 PMC9470293

[11] Claus EB Risch N Thompson WD . Genetic analysis of breast cancer in the cancer and steroid hormone study. Am J Hum Genet 1991; 48: 232–242. 1990835 PMC1683001

[12] Gail MH Brinton LA Byar DP , et al. Projecting individualized probabilities of developing breast cancer for white females who are being examined annually. J Natl Cancer Inst 1989; 81: 1879–1886. 2593165 10.1093/jnci/81.24.1879

[13] Costantino JP Gail MH Pee D , et al. Validation studies for models projecting the risk of invasive and total breast cancer incidence. J Natl Cancer Inst 1999; 91: 1541–1548. 10491430 10.1093/jnci/91.18.1541

[14] Mateo G F Granado-Font E Ferre-Grau C , et al. Mobile phone Apps to promote weight loss and increase physical activity: a systematic review and meta-analysis. J Med Internet Res 2015; 17: e253. 10.2196/jmir.4836 PMC4704965 26554314

[15] Carter MC Burley VJ Nykjaer C , et al. Adherence to a smartphone application for weight loss compared to website and paper diary: pilot randomized controlled trial. J Med Internet Res 2013; 15: e32. 10.2196/jmir.2283 PMC3636323 23587561

[16] Romeo A Edney S Plotnikoff R , et al. Can smartphone apps increase physical activity? Systematic review and meta-analysis. J Med Internet Res 2019; 21: e12053. 10.2196/12053 PMC6444212 30888321

[17] Coughlin SS Besenyi GM Bowen D , et al. Development of the physical activity and your nutrition for cancer (PYNC) smartphone app for preventing breast cancer in women. Mhealth 2017; 3: 5. 28293621 10.21037/mhealth.2017.02.02 PMC5344121

[18] Regmi K Kassim N Ahmad N , et al. Effectiveness of Mobile Apps for smoking cessation: a review. Tob Prev Cessat 2017; 3: 12. 32432186 10.18332/tpc/70088 PMC7232804

[19] Whittaker R McRobbie H Bullen C , et al. Mobile phone text messaging and app-based interventions for smoking cessation. Cochrane Database Syst Rev 2019; 10: CD006611. 10.1002/14651858.CD006611.pub5 PMC6804292 31638271

[20] Laurens MC Pieterse ME Brusse-Keizer M , et al. Alcohol avoidance training as a Mobile App for problem drinkers: longitudinal feasibility study. JMIR Mhealth Uhealth 2020; 8: e16217. 10.2196/16217 PMC7189254 32286235

[21] Harvey BJ Miller AB Baines CJ , et al. Effect of breast self-examination techniques on the risk of death from breast cancer. CMAJ 1997; 157: 1205–1212. 9361639 PMC1228347

[22] Blajda J Barnas E Kucab A . Application of personalized education in the mobile medical app for breast self-examination. Int J Environ Res Public Health 2022; 19: 4482. 35457349 10.3390/ijerph19084482 PMC9032731

[23] Heo J Chun M Lee KY , et al. Effects of a smartphone application on breast self-examination: a feasibility study. Healthc Inform Res 2013; 19: 250–260. 24523989 10.4258/hir.2013.19.4.250 PMC3920037

[24] Lee H Ghebre R Le C , et al. Mobile phone multilevel and multimedia messaging intervention for breast cancer screening: pilot randomized controlled trial. JMIR Mhealth Uhealth 2017; 5: e154. 10.2196/mhealth.7091 PMC5698632 29113961

[25] Google LLC. Developer content policy, https://play.google.com/intl/de/about/developer-content-policy/ (accessed 22.11. 2022).

[26] Apple Inc. Guidelines for developers, https://developer.apple.com/app-store/review/guidelines/ (accessed 22.11. 2022).

[27] Fahy E Hardikar R Fox A , et al. Quality of patient health information on the internet: reviewing a complex and evolving landscape. Australas Med J 2014; 7: 24–28. 24567763 10.4066/AMJ.2014.1900 PMC3920473

[28] Kuenzel U Monga Sindeu T Schroth S , et al. Evaluation of the quality of online information for patients with rare cancers: thyroid cancer. J Cancer Educ 2018; 33: 960–966. 28120139 10.1007/s13187-017-1173-z

[29] Chen R Santo K Wong G , et al. Mobile Apps for dental caries prevention: systematic search and quality evaluation. JMIR Mhealth Uhealth 2021; 9: e19958. 10.2196/19958 PMC7840287 33439141

[30] Scholz S Teetz L . Smart health via mHealth? Potentials of mobile health apps for improving prevention and adherence of breast cancer patients. Digit Health 2022; 8: 20552076221074127. 35096411 10.1177/20552076221074127 PMC8796094

[31] Tonsaker T Bartlett G Trpkov C . Health information on the internet: gold mine or minefield? Can Fam Physician 2014; 60: 407–408. 24828994 PMC4020634

[32] Mobasheri MH Johnston M King D , et al. Smartphone breast applications - what's the evidence? Breast 2014; 23: 683–689. 25153432 10.1016/j.breast.2014.07.006

[33] Teplinsky E Ponce SB Drake EK , et al. Online medical misinformation in cancer: distinguishing fact from fiction. JCO Oncol Pract 2022; 18: 584–589. 35357887 10.1200/OP.21.00764 PMC9377685

[34] Vergani L Marton G Pizzoli SFM , et al. Training cognitive functions using Mobile apps in breast cancer patients: systematic review. JMIR Mhealth Uhealth 2019; 7: e10855. 10.2196/10855 PMC6444278 30888326

[35] Kapoor A Nambisan P Baker E . Mobile applications for breast cancer survivorship and self-management: a systematic review. Health Informatics J 2020; 26: 2892–2905. 32842830 10.1177/1460458220950853

[36] Giunti G Giunta DH Guisado-Fernandez E , et al. A biopsy of breast cancer mobile applications: state of the practice review. Int J Med Inform 2018; 110: 1–9. 29331247 10.1016/j.ijmedinf.2017.10.022

[37] Moher D Liberati A Tetzlaff J , et al. Preferred reporting items for systematic reviews and meta-analyses: the PRISMA statement. Ann Intern Med 2009; 151: 264–269. 19622511 10.7326/0003-4819-151-4-200908180-00135

[38] Altmannshofer S Huebner H . Protocol for a systematic review comparing mobile health apps for breast cancer prevention and education. INPLASY protocol 202380101, 2023. doi:10.37766/inplasy2023.8.0101

[39] Stoyanov SR Hides L Kavanagh DJ , et al. Mobile app rating scale: a new tool for assessing the quality of health mobile apps. JMIR Mhealth Uhealth 2015; 3: e27. 10.2196/mhealth.3422 PMC4376132 25760773

[40] Bardus M van Beurden SB Smith JR , et al. A review and content analysis of engagement, functionality, aesthetics, information quality, and change techniques in the most popular commercial apps for weight management. Int J Behav Nutr Phys Act 2016; 13: 35. 26964880 10.1186/s12966-016-0359-9 PMC4785735

[41] Yang S Bui CN Park K . Mobile health Apps for breast cancer: content analysis and quality assessment. JMIR Mhealth Uhealth 2023; 11: e43522. 10.2196/43522 PMC9999256 36821352

[42] Escriche-Escuder A De-Torres I Roldan-Jimenez C , et al. Assessment of the quality of Mobile applications (Apps) for management of low back pain using the mobile App rating scale (MARS). Int J Environ Res Public Health 2020; 17: 20201209. doi:10.3390/ijerph17249209 PMC7763508 33317134

[43] Core Team R. R: A Language and Environment for Statistical Computing. Vienna, Austria: R Foundation for Statistical Computing, 2022.

[44] Musgrave LM Kizirian NV Homer CSE , et al. Mobile phone Apps in Australia for improving pregnancy outcomes: systematic search on App stores. JMIR Mhealth Uhealth 2020; 8: e22340. 10.2196/22340 PMC7704277 33196454

[45] Martin-Payo R Ferreras-Losilla L Gonzalez-Mendez X , et al. Apps for individuals diagnosed with breast cancer: a preliminary assessment of the content and quality of commercially available apps in spanish. Mhealth 2021; 7: 20210120. doi:10.21037/mhealth-19-191 PMC7882267 33634185

[46] Medizinprodukte B . Das fast-track-verfahren für digitale gesundheitsanwendungen (DiGA) nach § 139e SGB V. Ein Leitfaden für Hersteller, Leistungserbringer und Anwender. 22–24, 2023

[47] Bellanger M Barry K Rana J , et al. Cost-Effectiveness of lifestyle-related interventions for the primary prevention of breast cancer: a rapid review. Front Med (Lausanne) 2019; 6: 325. doi:10.3389/fmed.2019.00325 32117999 PMC7013088

[48] Carrello J Hayes A Baur LA , et al. Potential cost-effectiveness of e-health interventions for treating overweight and obesity in Australian adolescents. Pediatr Obes 2023; 18: e13003. 10.1111/ijpo.13003 PMC10909552 36649693

[49] Floch J Zettl A Fricke L , et al. User needs in the development of a health App ecosystem for self-management of cystic fibrosis: user-centered development approach. JMIR Mhealth Uhealth 2018; 6: e113. 10.2196/mhealth.8236 PMC5964302 29739742

[50] Sartori F Tonelli LL . Fuzzy personalization of mobile apps: a case study from mHealth domain. In: Cham, 2022. Heidelberg: Springer International Publishing, pp.91–108.

[51] Bauer AM Iles-Shih M Ghomi RH , et al. Acceptability of mHealth augmentation of collaborative care: a mixed methods pilot study. Gen Hosp Psychiatry 2018; 51: 22–29. 29272712 10.1016/j.genhosppsych.2017.11.010 PMC6512981

[52] Bendixen RM Fairman AD Karavolis M , et al. A user-centered approach: understanding client and caregiver needs and preferences in the development of mHealth Apps for self-management. JMIR Mhealth Uhealth 2017; 5: e141. 10.2196/mhealth.7136 PMC5635231 28951378

